# Cell cycle arrest of cardiomyocytes in the context of cardiac regeneration

**DOI:** 10.3389/fcvm.2025.1538546

**Published:** 2025-04-28

**Authors:** Qingling Xu, Xinhui Chen, Chunyige Zhao, Ying Liu, Jianxun Wang, Xiang Ao, Wei Ding

**Affiliations:** ^1^ Department of Comprehensive Internal Medicine, The Affiliated Hospital of Qingdao University, Qingdao, Shandong, China; ^2^ School of Basic Medicine, Qingdao University, Qingdao, Shandong, China; ^3^ Institute for Translational Medicine, The Affiliated Hospital of Qingdao University, Qingdao Medical College, Qingdao University, Qingdao, Shandong, China

**Keywords:** cardiomyocyte, proliferation, cardiac regeneration, cell cycle arrest, cardiovascular disease

## Abstract

The limited capacity of adult mammalian cardiomyocytes to undergo cell division and proliferation is one of the key factors contributing to heart failure. In newborn mice, cardiac proliferation occurs during a brief window, but this proliferative capacity diminishes by 7 days after birth. Current studies on cardiac regeneration focused on elucidating changes in regulatory factors within the heart before and after this proliferative window, aiming to determine whether potential association between these factors and cell cycle arrest in cardiomyocytes. Facilitating the re-entry of cardiomyocytes into the cell cycle or reversing their exit from it represents a critical strategy for cardiac regeneration. This paper provides an overview of the role of cell cycle arrest in cardiac regeneration, briefly describes cardiomyocyte proliferation and cardiac regeneration, and systematically summarizes the regulation of the cell cycle arrest in cardiomyocytes, and the potential metabolic mechanisms underlying cardiomyocyte cycle arrest. Additionally, we highlight the development of cardiovascular disease drugs targeting cardiomyocyte cell cycle regulation and their status in clinical treatment. Our goal is to outline strategies for promoting cardiac regeneration and repair following cardiac injury, while also pointing toward future research directions that may offer new technologies and prospects for treating cardiovascular diseases, such as myocardial infarction, arrhythmia and heart failure.

## 1 Introduction

Cardiovascular disease (CVD), especially ischemic heart disease, has become a major global public health challenge and remains the leading cause of mortality worldwide, despite considerable advancements in prevention and treatment of CVDs (1). The limited regenerative capacity of the adult mammalian heart contributes to the progression of almost all CVDs to heart failure (HF). Studies have found that the renewal rate of human cardiomyocytes decreases significantly with age. From 1% annual renewal at age 25, it gradually decreases to 0.45% at age 75 (2). This diminished regenerative capacity further exacerbates the progression of HF. Cardiomyocytes lost due to cardiomyopathic injury, such as myocardial infarction (MI), can't be sufficiently replenished through proliferation and division in the adult mammalian heart, resulting in functional impairment at the site of injury and the formation of irreversible fibrotic scars. This heightened vulnerability increases the heart's susceptibility to subsequent damage (3). Currently, the prevailing clinical strategy for treating CVDs focuses on delaying disease progression rather than promoting repair or regeneration. For patients with end-stage HF, cardiac organ transplantation remains the only effective treatment option. However, the scarcity of transplantable organs falls far short of meeting the substantial clinical demand. Therefore, elucidating the regenerative mechanisms of endogenous cardiomyocytes represents a critical avenue for advancing CVD treatment.

The conventional belief is that the adult mammalian heart is a terminally differentiated organ in which cardiomyocytes have limited ability for mitosis and regeneration. However, in 2011, Porrello et al. demonstrated that neonatal mice exhibit significant endogenous cardiomyocyte proliferation and cardiac regenerative potential during the first seven days of life (P7). Notably, when P7 mice underwent apicoectomy, no myocardial regeneration occurred at the site of injury, and irreversible fibrosis developed, indicating a loss of regenerative capacity beyond this critical period (4). Consequently, what factors restrict cardiomyocyte proliferation in mammals, such as in P7 mice, thereby limiting cardiac regeneration? Therefore, elucidating the regulatory mechanisms governing cardiomyocyte proliferation and identifying key molecular switches controlling their cell cycle progression represents a central focus of cardiac regeneration regeneration.

The loss of cardiac regenerative capacity in adult mammals primarily results from the cessation of the cell cycle in cardiomyocytes, effectively closing the window for proliferation. First, there is a marked shift in energy metabolism between the embryonic and adult hearts. Specifically, embryonic cardiomyocytes predominantly rely on anaerobic glycolysis as their primary energy source, whereas adult cardiomyocytes primarily utilize oxygen-dependent mitochondrial oxidative phosphorylation (5). Second, the postnatal transition from anaerobic to aerobic respiration triggers an increase in reactive oxygen species (ROS) production and activates the DNA damage response pathway (DDR), ultimately leading to cell cycle arrest and maturation of cardiomyocytes (5–7). This raises the critical question of whether cell cycle withdrawal represents a cause or consequence of the diminished cardiac regenerative capacity. Taken together, these findings suggest that cell cycle activity plays a pivotal role in determining cardiac regenerative capacity.

In this paper, we comprehensively review the regulation of the cell cycle in cardiac regeneration, along with the regulators and mechanisms underlying myocardial cell cycle arrest that have been reported in recent years. Furthermore, we summarize cardiac regeneration strategies targeting the myocardial cell cycle, which provide innovative insights for cardiac regeneration and repair research.

## 2 Cardiomyocyte proliferation and cardiac regeneration

### 2.1 Source of neonatal cardiomyocytes

**Figure 1:**
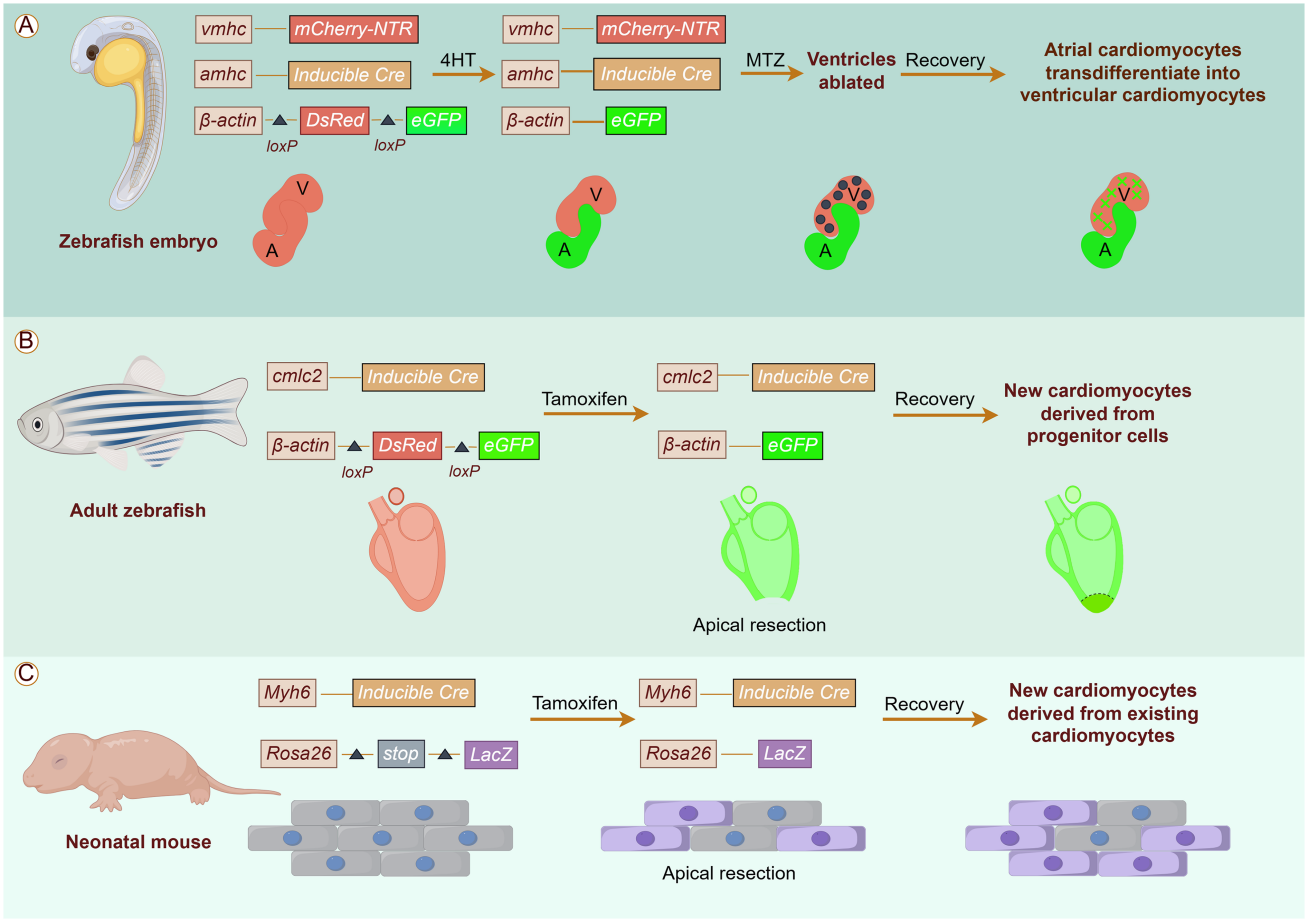
Origins of cardiomyocytes in cardiac regeneration. The potential sources of cardiomyocytes include **(A)** Transdifferentiation from other cell types; **(B)** Differentiation of progenitor cells; **(C)** Differentiation of progenitor cells.

Cardiac regeneration has garnered substantial attention, with a central scientific question being the origin of newborn cardiomyocytes. Current research proposes potential sources, including the transdifferentiation from other cell types and the differentiation of progenitor cells, and dedifferentiation of pre-existing cardiomyocytes (Figure 1).

The zebrafish heart has an amazing ability to regenerate throughout its life, thanks largely to the fact that its cardiomyocytes never fully exit the cell cycle and are able to proliferate in response to signals of injury to replenish lost myocardium (8). Fate mapping studies in zebrafish heart regeneration have demonstrated that fish replace lost cardiomyocytes primarily through the proliferation of undifferentiated cardiac precursor cells. By utilizing an EGFP dual fluorescent reporter gene driven by the cmlc2 promoter, researchers successfully traced the origin of newborn cardiomyocytes. Prior to cardiac injury, all cmlc2-expressing cardiomyocytes were labeled by eGFP expression, and most regenerating tissues exhibited eGFP expression 30 days after ventricular resection. These findings indicate that the newborn myocardium is predominantly derived from progenitor cells that subsequently differentiate into proliferative cardiomyocytes (9, 10). In recent years, studies have shown that transdifferentiation also contributes to cardiac regeneration. For instance, Zhang et al. discovered that differentiated atrial cardiomyocytes can transdifferentiate into ventricular cardiomyocytes using a combination of genetic fate mapping mapping and ventricle-specific genetic ablation system in zebrafish models. Notably, inhibition of Notch signaling blocked atrial-to-ventricular transdifferentiation and impaired cardiac regeneration (11). In addition, Poss et al. utilized a lineage-tracing method to fluorescently label zebrafish cardiomyocytes, confirming that neonatal cardiomyocytes are derived from pre-existing ones (12).

The switching of growth patterns in the mammalian heart during cardiac maturation is tightly controlled. Thus, mammalian cardiomyocytes regenerated after neonatal injury are derived from pre-existing cardiomyocytes, utilizing their proliferative activity that has not been completely lost, rather than transdifferentiated or induced stem cells (13). For example, Koudstaal et al. found by gene genealogical tracing techniques that cardiomyocytes can replenish damaged myocardium by de-differentiating and re-entering the cell cycle after injury in the neonatal mouse heart (14). The viewpoint was further substantiated by investigations into neonatal rat heart regeneration (4).

Overall, these investigations not only provide compelling evidence for cardiac plasticity during myocardial injury but also reveal multiple potential sources of cardiac resident cells for cardiac regeneration.

### 2.2 Cardiac regeneration in low-vertebrates and mammals

Lower vertebrates demonstrate superior cardiomyocyte regeneration capabilities compared to mammals. Zebrafish have exhibited successful regeneration and functional recovery in various cardiac injury models, including apical resection (AR), cryoinjury, and genetic cell ablation (12, 15–18). Amongst low-vertebrate species, tailless amphibians are also renowned for their remarkable cardiac regeneration capabilities. Following cardiac injury in salamanders, complete restoration of cardiac function without scarring can be achieved within three months (19–21). Additionally, it has been observed that adult frog cardiomyocytes limited some proliferative capacity and the ability to dedifferentiate. However, scar tissue formation occurs after cardiac injury, preventing complete cardiac regeneration (22).

The most significant difference between mammals and lower vertebrates lies in their capacity to complete the cell cycle (23, 24). In contrast to haploid cardiomyocytes, polyploid cardiomyocytes exhibit restricted proliferative capacity and typically fail to progress through the normal cell cycle (25). Furthermore, it has been demonstrated that mononuclear diploid cardiomyocytes play a crucial role in cardiac regeneration and repair due to their substantial proliferative potential (26). Unlike zebrafish mononuclear cardiomyocytes, which can re-enter the cell cycle during adulthood, adult mammalian cardiomyocytes transition from proliferative mononuclear diploid cells to nonproliferating binucleated diploid cells either before (27) or shortly after birth (28), thereby limiting their proliferative capacity (28, 29). Whether diploid or polyploid cardiomyocytes possess proliferate capabilities during cardiac regeneration and contribute to myocardial repair, as well as offering therapeutic strategies remains an area requiring further investigation.

Unlike zebrafish, adult mammalian hearts exhibit limited cardiac regeneration following injury (30). However, studies of neonatal cardiac regeneration in mice have demonstrated robust regenerative responses in injured areas after AR and MI surgery (4, 31). These findings highlight a specific time window during which the mammalian heart displays a strong regenerative response immediately after birth. However, Robledo et al. reported that the rat heart fails to achieve complete regeneration when subjected to burn injury 4–7 days after birth, underscoring the need for further investigation into the mechanisms underlying the differences in regenerative capacity between neonatal rats and mice (32). Interestingly, AR surgery performed on mice seven days after birth did not trigger a significant regenerative response (4), indicating that the heart's regenerative potential significantly diminishes shortly after birth. Recent reports on neonatal corrective cardiac surgery (33) and neonatal MI (34) suggest that human neonates may possess a higher regenerative capacity, enabling them to recove cardiac function effectively.

In the context of cardiac regeneration, lower vertebrates such as zebrafish exhibit complete restoration of the heart following injury, whereas mammals lose this regenerative capacity shortly after birth. Thus, what factors restrict cardiomyocyte proliferation, thereby reducing the regenerative potential in mammals? Consequently, elucidating the regulatory mechanisms that govern cardiomyocyte proliferation and identifying key regulators that orchestrate cardiac regeneration remain crucial objectives in cardiac regeneration research.

Overall, there are significant differences in the origin of neonatal cardiomyocytes and the regenerative capacity of the heart between lower vertebrates and mammals. Nevertheless, these studies provide an important theoretical foundation for the advancement of cardiac regenerative medicine. The development of cardiac regenerative medicine will emphasize multidisciplinary collaboration and innovation, integrating cell therapy, gene therapy, tissue engineering, and biomaterials to achieve effective treatment after MI. Future research will need to further optimize these techniques, address existing challenges, and facilitate the broader application of regenerative medicine in MI treatment.

## 3 Modulation of cell cycle on cardiomyocyte proliferation

**Figure 2:**
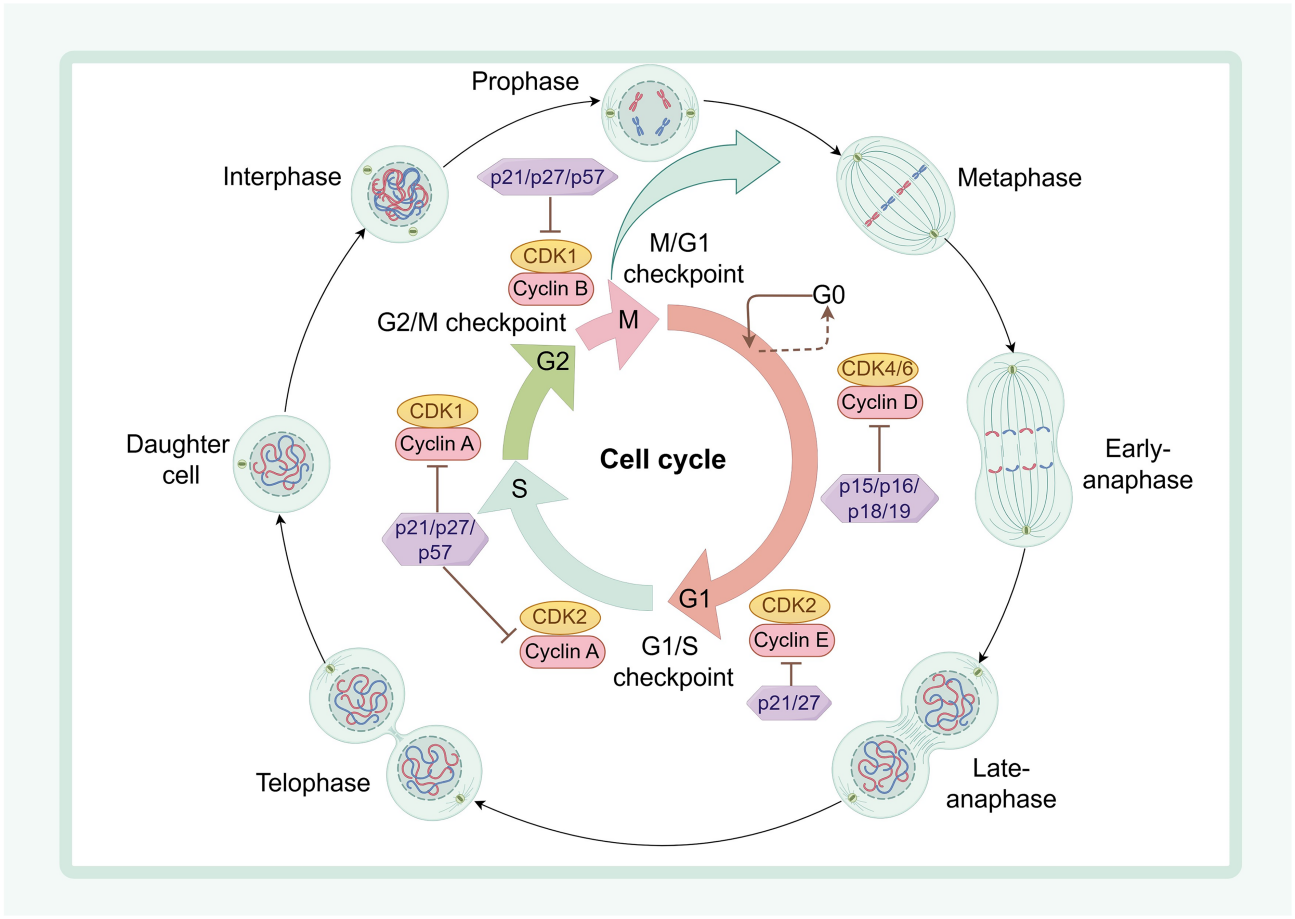
The regulatory mechanism of cell cycle in cardiac regeneration. Cyclin D interacts with CDK4/6 to initiate entry into G1 phase; late G1 CyclinE synergizes with CDK2 to enter S phase; CDK2, CDK1, and Cyclin A coordinate with each other to drive progression through S and G2 phases, respectively; and Cyclin B interacts with CDK1 to drive progression from late G2 to the end of mitosis. In contrast, two inhibitor families, the INK4 family (p15, p16, p18, and p19) and the WAF1/CIP/KIP family (p21, p27, and p57), interact with CDK to arrest the cell cycle. Cytoplasmic divisions are divided into interphase, prophase, metaphase, anaphase and telophase.

Cell proliferation depends on the cell cycle, a complicated process that spans from the generation of daughter cells through one round of division to the subsequent preparation for new rounds of division. At the molecular level, this process is synergistically regulated by specific cellular cyclins (Cyclin) and cyclin-dependent kinases (CDKs) (35) (Figure 2).

The activation of CDKs is dependent on the dynamic regulation of their associated cyclins (36). Notably, CDKs and specific cyclins play distinct roles during different phases of the cell cycle. Specifically, CDK1, CDK2, CDK4, and CDK6 are crucial for cell cycle progression (37–39). For instance, growth factors promote entry into the G1 phase, activate Cyclin D1, D2, and D3, and subsequently interact with CDK4 or CDK6 to drive cell cycle progression (40–42). It has been reported that overexpression of cyclin D2 in mice following MI induces DNA synthesis and cardiomyocyte proliferation while reducing infarct size (43). Furthermore, CDK1 and Cyclin B regulate progression from late G2 to the end of mitosis (38, 44). Overexpression of the Cyclin B1 complexed with cell division cycle kinase 2 (CDC2) facilitates cardiomyocyte division in adult mice, whereas deletion of this complex leads to G2/M phase arrest and inhibits proliferation in adult mouse cardiomyocytes (45). Additionally, overexpression of Cyclin A2 induces DNA synthesis and cardiomyocyte mitosis, thereby enhancing cardiac function in ischemically injured mice (46).

CDK-Cyclin activity is regulated by cyclin-dependent protein kinase inhibitory proteins (CKIs). A combination of four cell cycle regulators, CDk1 with CCNB and Cdk4 with CCND, activates cardiomyocyte proliferation. Furthermore, the use of Wee1 inhibitors in conjunction with TGF-β inhibitors promotes cardiomyocyte proliferation, reduces fibrosis area after cardiac injury, and improves cardiac function (47). Recent studies have revealed that adult mouse cardiomyocytes harbor multiple negative regulators in the form of CKI proteins, which restrict the cycling activity of these cells in adult mice, thereby suppressing their proliferative and division capabilities (48).

Changes in postnatal mammalian nutrient metabolism and ingestion lead to increased production of ROS and activation of the DDR (5, 6), both of which result in aberrant transcription of CDKs-Cyclin, leading to uncontrolled mitosis and cell cycle arrest in cardiomyocytes (49, 50). Modulating cell cycle regulators during cardiac growth and development to influence cardiomyocyte proliferation, thereby promoting cardiac regeneration, is anticipated as a prospective therapeutic strategy for cardiac repair.

In summary, cell cycle regulation is the key to cardiomyocyte proliferation, and future studies will explore its mechanism in depth through various strategies, such as gene therapy, metabolic intervention, cell transplantation and tissue engineering, and develop effective therapeutic means to promote myocardial regeneration and repair.

## 4 Regulators of cell cycle arrest in cardiomyocytes

**Figure 3:**
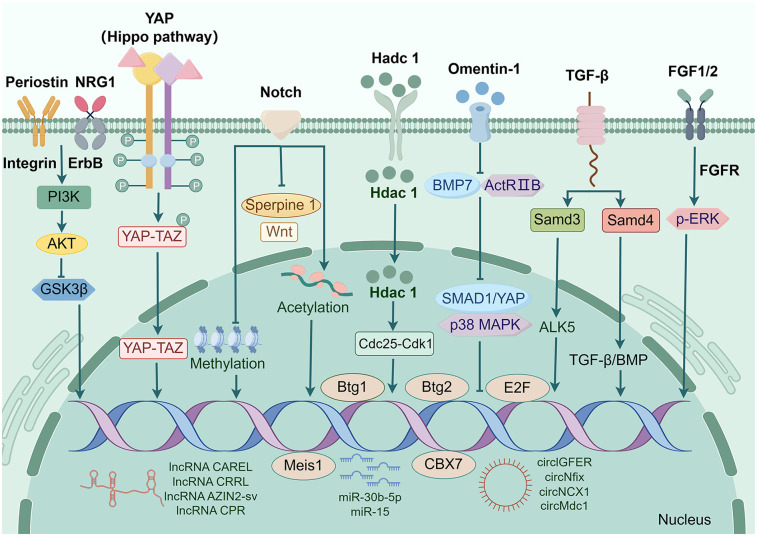
Factors that induce myocardial cell cycle arrest. Transcription factors including Btg1, Btg2, E2F, MEIS1, etc; Secretion factors including Hdac1, Omentin1, Periostin, NRG1, etc.; Signaling pathways including TGF-β pathway, Hippo pathway, Notch pathway, etc.; Non-coding genes including circGFER, circNCX1, miR-30b-5p, miR-15, lnc RNA CAREL, lnc RNACPR, etc.

Currently, an increasing number of studies in cardiac regeneration models have identified factors that inhibit cardiomyocyte proliferation by arresting cell cycle progression. These factors primarily include cell cycle proteins, coding and non-coding genes, as well as signaling pathways (Figure 3).

### 4.1 Coding genes

In recent years, a multitude of coding genes that regulate the cell cycle in cardiac regeneration have been identified by researchers. These factors arrest the cardiomyocyte cell cycle through diverse pathways, thereby blocking both cardiomyocyte proliferation and cardiac regeneration (51).

Certain cell cycle regulators can suppress the proliferation of cardiomyocytes by directly binding to Cyclin or indirectly activating CDKs. For instance, the transcription factor E2F interacted with Cyclin to regulate both the proliferation and differentiation of cardiomyocytes (52). Deletion of E2F1, E2F2, and E2F3 has been shown to prevent cells from entering the S phase, thereby arresting the cell cycle of cardiomyocytes and inhibiting their capacity for proliferation (53). In 2013, Sadek et al. discovered that a transcription factor Meis1 played a crucial role in preventing heart muscle cells from dividing (54). Initially thought to act alone by Sadek's team during early studies where they knocked out its encoding gene in mice resulting in prolonged window for neonatal mouse cardiomyocyte division. However this prolongation was short-lived as these deficient cardiomyocytes eventually slowed down their division and ceased proliferating (54). Subsequently identified was Hoxb13 as a chaperone protein for Meis1; when both Meis1 and Hoxb13 were knocked out together in mice models it caused regression of cardiomyocytes back to an earlier developmental stage characterized by decreased size but increased number (54). Furthermore, they found that the CDK inhibitors p15, p16, and p21 are essential for transcriptional activation of Mesi1 as its transcriptional activation targets (55).

A study by Velayutham et al. revealed that the expression patterns of transcriptional co-regulators Btg1 and Btg2 during neonatal mouse cardiac development were consistent with the observed pattern of cell cycle of cardiomyocytes arrest. Btg1/2 constitutive double knockout cardiac tissues exhibited an increase in pH3^+^ mitotic cardiomyocytes at P7 but not at P30 (56). This finding indicates that suggest that Btg1 and Btg2 are novel factors involved in postnatal cell cycle of cardiomyocytes arrest. Zinc finger transcription factors are closely associated with cardiomyocyte proliferation. Mohammadi et al. demonstrated that specific knockdown of GATA4 in cardiomyocytes was found to reduce cardiomyocyte proliferation and myocardial angiogenesis in a rat model of cryoinjury, suggesting impaired cardiac regenerative capacity (57). CBX7 levels in the heart exhibit a continuous increase after birth and persist throughout adulthood. Overexpression of CBX7 in neonatal mouse cardiomyocytes has been shown to inhibit proliferation and promote multinucleation (58).

Secreted factors play important roles in cardiomyocyte cycle arrest and cardiac regeneration. Yang et al. identified omentin-1 as an adipokine that directly interacts with bone morphogenetic protein 7 (BMP7), regulating cardiomyocyte maturation. Omentin-1 prevents BMP7 from binding to activin type II receptor B (ActRIIB), subsequently inhibiting downstream signaling pathways involving DPP homolog 1 (SMAD1)/yes-associated protein (YAP) and p38 mitogen-activated protein kinase (p38 MAPK) (59). In another study, p38α negatively regulated adult zebrafish cardiomyocyte proliferation (60). Interestingly, Wu et al. showed that endogenous BMP signaling in zebrafish induced cell cycle arrest, thereby attenuating cardiomyocyte dedifferentiation and ultimately impairing myocardial regeneration (61). In a zebrafish regeneration model study, deficiency of histone deacetylase 1 (Hdac1) was found to affect the Cdc25-Cdk1 cell cycle axis, resulted in impaired progression through the G2/M phase and Cell cycle of cardiomyocytes arrest (62). Periostin, a secreted extracellular matrix protein, induces reentry into the cell cycle of already differentiated cardiomyocytes through the PI3K pathway, reduces post-infarction fibrosis, decreases infarct size, promotes angiogenesis and improves cardiac function (63). In addition, Chen et al. showed that Periostin mediates cardiomyocyte proliferation after myocardial infarction through PI3K/AKT/GSK3β signaling (64). NRG1, an epidermal growth factor, stimulates cardiomyocytes to re-enter the cell cycle and divide by activating downstream signaling pathways through its receptors ErbB2, ErbB3, and ErbB4 (65). However, although NRG1 transiently activates ErbB receptors to promote cardiomyocyte proliferation, it may inhibit overproliferation in adult myocardium through a negative feedback mechanism (66). Fibroblast growth factor FGF belongs to the family of secreted proteins. Studies have shown that activation of the FGF signaling pathway promotes cardiomyocyte proliferation and hypertrophy. For example, FGF10 regulates cell cycle reentry in cardiomyocytes by interacting with FGFR2b (embryonic stage) and FGFR1b (adult stage), thereby increasing ventricular wall thickness (67). However, chronic activation of the FGFR1 signaling pathway leads to cardiomyocyte hypertrophy and cardiac remodeling as evidenced by increased cardiomyocyte cross-sectional area, interstitial fibrosis, and disturbed myocyte alignment (68). In summary, secreted factors regulate cardiomyocyte cycle arrest through a complex network, a property that maintains cardiac homeostasis while limiting regenerative capacity. Future studies need to further resolve the spatiotemporal-specific roles of specific factors under pathological/physiological conditions to develop targeted intervention strategies.

Manipulating factors that induce cell cycle arrest during cardiac growth and development to influence cardiomyocyte proliferation, thereby promoting cardiac regeneration and repair, is anticipated as a prospective therapeutic strategy for cardiac restoration.

### 4.2 Non-coding genes

In recent years, an increasing number of evidence has demonstrated the involvement of noncoding RNAs (ncRNAs) in cardiac regeneration and their key roles in the pathogenesis of various CVDs. Specifically, circular RNAs (circRNAs) have emerged as crucial drivers of Cell cycle of cardiomyocytes arrest during adulthood. The Meis1-driven expression of circNfix exerts an inhibitory effect on cardiomyocyte proliferation. circNfix promoted the ubiquitin-dependent degradation of Y-box binding protein 1 (Ybx1), thereby suppressing the expression of Yap1 target genes Cyclin A2 and Cyclin B1, resulting in the inhibition of cardiomyocyte proliferation (69). Silencing of circMdc1 induced by oxidative stress allows reentry into the cell cycle of cardiomyocytes (70). Moreover, by competitively binding to proteins required for translation of its host gene, Mdc1, circMdc1 influenced oxidative DNA damage and controlled the window for cardiomyocyte cell cycle progression (70). In our previous work, we verified that circNCX1 plays a role in regulating cardiomyocyte proliferation by negatively regulating ubiquitination of the transcriptional activator BRG1 (71). The cyclic insulin circlGFIR interacted with miR-5-362p in the adult heart to inhibit cardiomyocyte proliferation, providing a crucial experimental basis for cardiac regeneration regulation (72). Long noncoding RNAs (lncRNAs) play a pivotal role in maintaining cellular homeostasis. Wang et al. discovered that the lncRNA CPR was an effective repressor of cardiomyocyte proliferation, offering a potential lncRNA-based therapeutic target for cardiac repair and regeneration (73). Furthermore, lncRNAs CAREL, CRRL, and AZIN2-sv have been shown to inhibit cardiomyocyte proliferation by interacting with micro RNAs (miRNAs) (74–76). MiRNAs are crucial regulators in cardiac development and involved in modulating various CVDs. In hearts of rats with MI, 85 differentially expressed miRNAs were identified, among which miR-30b-5p was down-regulated. Treatment of its inhibitor enabled reentry into the Cell cycle of cardiomyocytes while inhibiting cardiomyocyte apoptosis (77). Furthermore, cardiac-specific overexpression of miR-128 impaired cardiomyocyte proliferation and cardiac function, whereas miR-128 deletion inhibited p27 expression by enhancing SUZ12 expression, which further activated Cyclin E and CDK2 and contributed to cardiomyocyte cycle re-entry (78). The miR-15 family plays an important negative regulatory role in cardiomyocyte proliferation and cardiac regeneration. It has been shown that miR-15 family members (e.g., miR-195) are upregulated in the postnatal heart and promote cardiomyocyte proliferative arrest by repressing the expression of the cell cycle-associated gene Chek1 (79). chek1 plays a critical role in the G2/M phase transition and mitotic progression, and its expression is inhibited by the miR-15 family, leading to cardiomyocyte proliferation arrest (79). Meanwhile, the proliferative capacity of adult cardiomyocytes could be activated by inhibition of miR-15 family members and improved cardiac function in a myocardial infarction model (80). Thus, by inhibiting miR-15 family members, the proliferative capacity of cardiomyocytes can be activated, thus providing a new strategy for cardiac regeneration. These findings provide an important theoretical basis for the development of miRNA-based therapies for cardiac regeneration.

Collectively, these findings suggest that targeting ncRNA molecules holds great promise as a therapeutic strategy to promote cardiac repair processes and provides valuable insights for further investigations into the molecular mechanisms underlying cardiac regeneration.

### 4.3 Signaling pathways

#### 4.3.1 Hippo-YAP signaling pathway

The Hippo signaling pathway plays a crucial role in regulating cardiomyocyte proliferation and cardiac regeneration (81). Heallen et al. demonstrated that Hippo signaling prevents postnatal re-entry of the cell cycle in cardiomyocytes, thereby inhibiting cardiomyocyte renewal and regeneration in adult mammals (82). YAP is highly activated in the heart before birth. However, its expression is suppressed after birth due to activation of Hippo signaling, consequently restraining cardiomyocyte proliferation (83). Consistent with this, overexpression of YAP during the postnatal period promotes the proliferation of postmitotic cardiomyocytes (83, 84). Collectively, these findings indicate that as cardiomyocytes progressively exit the cell cycle after birth, there is a substantial increase in Hippo signaling activity accompanied by a significant decrease in YAP activity. Therefore, targeting the Hippo-YAP signaling pathway has emerged as a promising strategy for achieving adult heart regeneration.

The role of the Hippo-YAP signaling pathway in cardiac injury has also been extensively investigated. Heallen et al. demonstrated that blockade of Hippo signaling through deletion of Sav1 or Lats1/2 facilitated cardiac regeneration in neonatal suckling mice subjected to AR and MI (82). However, depletion of Salvador, a key component of the Hippo pathway, in the hearts of mice with ischemic HF three weeks post-MI resulted in enhanced angiogenesis, reduced fibrosis, and restored cardiac function, ultimately promoting partial regeneration of the myocardium (85). These findings suggest that targeting the Hippo-YAP signaling pathway may reverse HF progression and provide therapeutic benefits for HF patients. Furthermore, adeno-associated virus subtype 9 (AAV9)-mediated overexpression of human YAP in the hearts of adult mice following MI significantly stimulated cardiomyocyte proliferation, improved cardiac function, and increased survival rates (86). Therefore, gene therapy utilizing AAV delivery vectors holds promise as a strategy for modifying outcomes after MI.

Our previous study demonstrated that YAP plays a crucial role in cardioprotection by targeting Parkin to suppress cardiotoxicity induced by the antitumor agent adriamycin (87). Furthermore, we confirmed the regulatory function of Parkin in cardiomyocyte necrosis (88). Based on these findings, we hypothesized Parkin may be involved in regulating cardiomyocyte cell cycle arrest through YAP signaling, thereby influencing cardiomyocyte proliferation and regeneration.

#### 4.3.2 Notch signaling pathway

The Notch signaling pathway is a highly conserved signaling pathway that plays a critical role in cardiomyocyte proliferation and cardiac development (89). Early study on the zebrafish heart models revealed that msxB/C genes were significantly upregulated during cardiac regeneration, while the expression of Notch1 was notably increased prior to Msx activation, indicating early activation of the Notch pathway preceding zebrafish heart regeneration (90). Subsequent investigation focused on embryonic heart regeneration in zebrafish using a ventricle-specific gene ablation system. Notch signaling was activated in the atrial endocardium following ventricular ablation. Moreover, inhibition of Notch signaling blocked the conversion of atrial cardiomyocytes into ventricular cardiomyocytes, thereby affecting heart regeneration (11). In an adult zebrafish model of heart regeneration, Zhao et al. demonstrated that downregulation of Notch expression in the endocardium following ventricular amputation inhibited cardiomyocyte proliferation and triggered fibrosis. Upon injury to zebrafish hearts, Wnt signaling was activated, impairing cardiomyocyte proliferation and promoting scar formation. However, inhibition of the Wnt pathway partially restored the proliferation of Notch-suppressed cardiomyocytes in the endocardium (91), suggesting that Notch signaling promotes cardiomyocyte proliferation by inhibiting myocardial Wnt activity. Additionally, in a cryoinjury model of zebrafish hearts, it was observed that Serpine1, another endocardial molecule, exhibited strong expression during early stages after cryoinjury when Notch signaling was inhibited. However, its expression gradually decreased during later stages. These findings indicate that Notch signaling regulates endocardial maturation and cardiomyocyte proliferation by inhibiting Serpine1 (92).

In recent years, there has been an increasing attention on the investigation of Notch signaling in the mammalian heart. Collesi et al. were the first to observe a high expression level of Notch1 in immature ventricular cardiomyocytes, which decreased with age. Overexpression of Notch or activation of the Notch signaling pathway using the ligand Jagged1 significantly stimulated proliferative signaling and promoted cardiomyocyte proliferation in suckling mice (93). However, overexpressing Notch1 using adeno-associated viral vectors was largely ineffective in stimulating cardiac repair after MI in adult mice. Subsequent research revealed that DNA methylation modifications within promoter regions of genes targeted by Notch signaling in adult cardiomyocytes inhibited Notch activation (94). Additionally, acetylation modifications occurred in proliferating neonatal rat cardiomyocytes, thereby maintaining the stability of Notch signaling. Overexpression of Sirt1, a negative regulator of Notch1, reversed its acetylation level and decreased its stability, thus closing the proliferation window of cardiomyocytes. Viral vectors were utilized to enhance the expression of acetylation-modified Notch1, resulting in enhanced cardiac regenerative capacity following AR in suckling mice (95). Knockdown of Notch1 suppressed pathways involved in cardiomyocyte cell cycle progression and mitosis, leading to proliferation defects in early-stage human cardiomyocytes. Single-cell transcriptome analysis revealed that epicardial and second ventricular progenitor cells differentiated at the expense of first ventricular progenitor cells, suggesting that Notch1 inhibits second ventricular histiocyte proliferation under normal conditions (96).

#### 4.3.3 TGF-β signaling pathway

The transforming growth factor-β (TGF-β) pathway plays a critical role in cardiac development and cardiomyocyte proliferation (97). Activation of TGF-β signaling primarily occurs through the Samd-dependent pathway. It has been demonstrated that the intracellular factor Smad4 mainly mediated TGF-β/BMP signaling. Specific knockdown of the Smad4 gene in the embryonic heart using the Cre-LoxP system resulted in a significant reduction in cardiomyocyte proliferation, severe defects in cardiac morphology, and stage-specific embryonic mortality. Furthermore, deletion of Smad4 affects the expression of TGF-β/BMP ligand, thereby disrupting its signaling cascade and ultimately impairing cardiac development while inhibiting embryonic cardiomyocyte proliferation (98). Smad3, a crucial marker of TGF-β signaling, induces the expression of TGF-β type I receptor ALK5 and initiates cardiomyocyte signaling in the injured region following cryoinjury of the zebrafish heart, thereby promoting cardiac regeneration. Inhibition of ALK5 using specific inhibitors reverses this process and completely impedes cardiac regeneration (99). Typically, TGF-β signaling activates ALK5 by recruiting type I receptors through type II receptors on the cell membrane for transphosphorylation. Moreover, in MI models, inhibition of ALK5 was found to attenuate Smad2 activation (100).

The activation of TGF-β signaling in cardiac regeneration can also be mediated by Samd-independent pathways. Although TGF-β can activate a wide range of signaling pathways, including the Rho/ROCK, p38 MAPK, and small GTPase pathways (101–104), little is currently known about the specific roles of these pathways in cardiac regeneration. Therefore, this represents an important future direction for research in cardiac regeneration.

In summary, regulators of cell cycle block in cardiomyocytes play a key role in cardiac regeneration. Future studies will deeply explore the regulatory mechanisms of cardiomyocyte proliferation through various strategies such as targeted therapy, gene editing, single-cell histology technology and improvement of the microenvironment, so as to provide new directions and methods for the development of cardiac regenerative medicine.

## 5 Mechanisms of metabolic regulation of cell cycle of cardiomyocytes blockade

**Figure 4:**
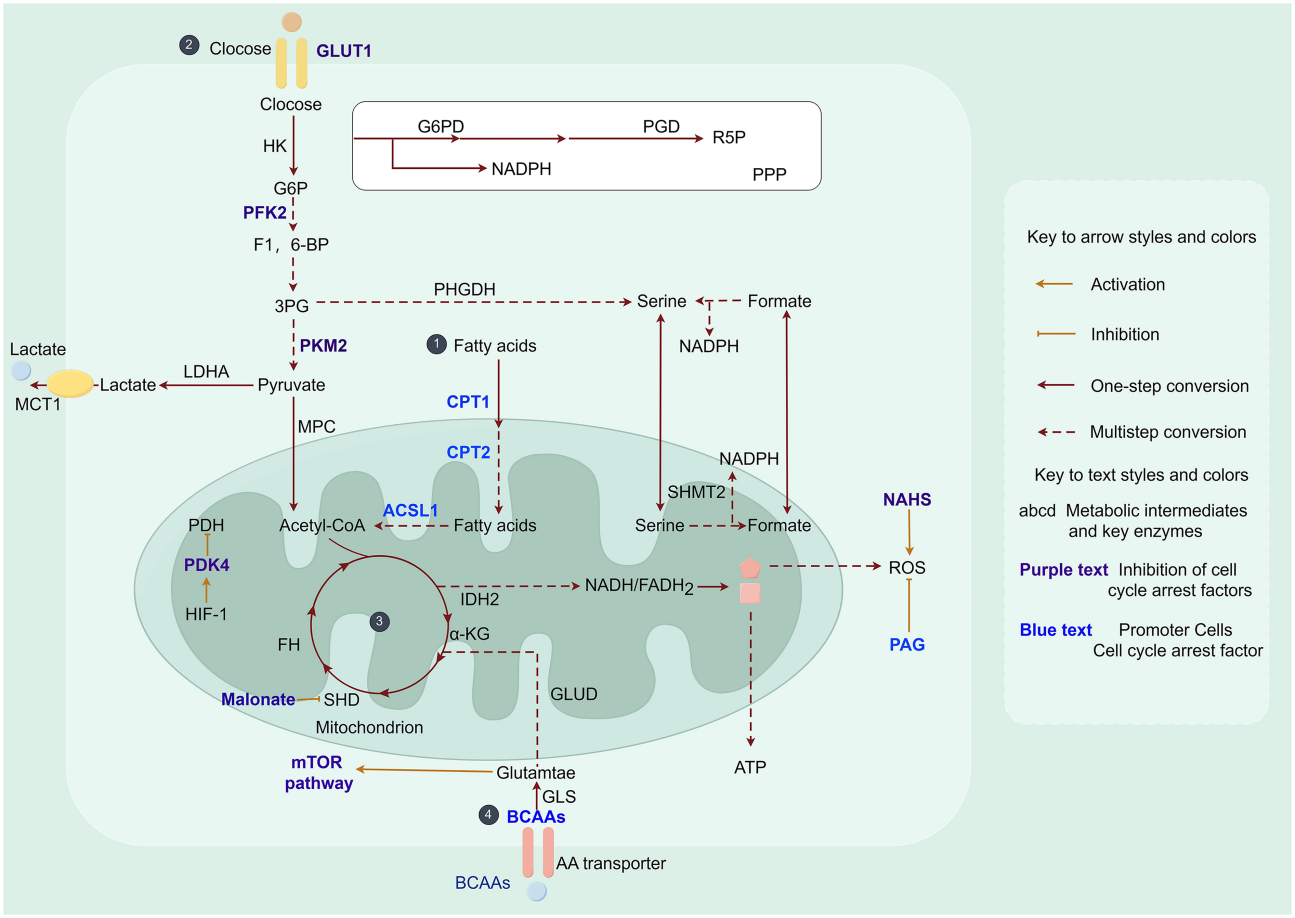
Metabolic pathways in myocardial cell cycle arrest. ① Fatty acid metabolism: ACSL1, acyl coenzyme A synthetase long chain family member 1; CPT, carnitine palmitoyltransferase. ② Glucose metabolism: GLUT, glucose transporter protein family; G6P, glucose-6-phosphate; PFK, phosphofructokinase; F1, 6-BP, 1, 6-bisphosphofructose; 3PG, 3-phosphoglycerate; PKM2, M2-pyruvate kinase; PDK, pyruvate dehydrogenase kinase; PDH, pyruvate dehydrogenase. ③ Tricarboxylic acid cycle: α-KG, α-ketoglutarate; SDH, succinate dehydrogenase; FH, fumarate hydratase; PAG, propylglycine; ROS, reactive oxygen species; NaHS, sodium bicarbonate. ④ Amino acid metabolism: BCAAs, branched-chain amino acids; mTOR, mammalian target of rapamycin. Acetyl-CoA is an intermediate product linking the three major nutrient metabolisms (fatty acid metabolism, glucose metabolism, and amino acid metabolism).

Recently, changes in metabolic patterns during cardiac regeneration have become a hot research topic. In mammals, the fetal period is characterized by relative hypoxia, and cardiac energy is primarily derived from glycolysis (105–108). Seven days after birth, as cardiac energy demand increases, glycolysis gradually decreases and is replaced by a significant increase in fatty acid oxidation to maintain energy metabolism in cardiomyocytes (107). Consequently, this transition from glycolysis to oxidative metabolism leads to an elevation in mitochondrial ROS production and DDR, ultimately resulting in cell cycle arrest (5, 6). Collectively, alterations in oxygen levels at different developmental stages influence changes in the heart's energy source. Furthermore, modifications in metabolic patterns impact the state of the cardiomyocyte cell cycle and subsequently affect its proliferative capacity (Figure 4).

### 5.1 Fatty acid metabolism induces cardiomyocyte cell cycle arrest

The adult heart primarily derives its energy from the oxidation of fatty acids (109). Among all energy substrates, fatty acids produce the highest amount of ATP per 2 carbon units but also have the greatest oxygen demand. Consequently, fatty acids are considered the least metabolically efficient cardiac energy substrate in terms of total ATP produced/oxygen consumed (110).

Studies have shown that inhibition of fatty acids promotes cardiomyocyte proliferation and cardiac regeneration (111, 112). Neonatal mice fed with fatty acid-deficient milk exhibited an extended postnatal window for cardiomyocyte proliferation, but eventually progressed to cell cycle arrest (111). Carnitine palmitoyltransferase 1 (CPT1), a crucial enzyme in fatty acid oxidation, plays an indispensable role in regulating the metabolic switch from glycolysis to fatty acid oxidation. Li et al. demonstrated that inactivation of the b-type subunit of CPT1 (CPT1b) abolished fatty acid oxidation in cardiomyocytes, inducing a transition from a mature to an immature metabolic state and thereby reversing cardiomyocyte cell cycle arrest (112). Furthermore, etomoxir (ETO), an inhibitor of CPT1 activity, was found to suppress fatty acid oxidation and stimulate cardiomyocyte proliferation and cardiac regeneration in neonatal mice (113). Inhibition of carnitine palmitoyltransferase 2 (CPT2), another key enzyme in fatty acid oxidation, promoted cardiomyocyte proliferation in 2-week-old mice (114). Acyl coenzyme A synthetase long-chain family member 1 (ACSL1) is an essential regulator of lipid metabolism. Its expression level progressively increases after birth. Knockdown of ACSL1 significantly enhanced cardiac regeneration in primary mouse cardiomyocytes and efficiently restored cardiac function and muscle regeneration in adult mice following MI (115).

Phosphatidic acid plays a crucial role in lipid anabolism. Cao et al. unveiled the involvement of sphingolipid metabolism in cardiac regeneration, where SphK1 and SphK2, the two isozymes responsible for producing sphingosine-1-phosphate, exhibiting contrasting roles in repairing neonatal cardiac injury (116). Therefore, it is reasonable to speculate that ketoester metabolism also plays an indispensable role in regulating cardiac regeneration. Taken together, targeting fatty acid utilization in cardiomyocytes enhances proliferation and represents a promising therapeutic target for cardiac regenerative therapies (Table 1).

**Table 1:** Metabolic pathways in cardiomyocyte cell cycle arrest.

Metabolic pathway	Species	Intervention targets	Key messages	References
Fatty acid oxidation	Postnatal mice	Fatty acid	Prolonged cardiomyocyte proliferation window in mice fed fatty acid-deficient milk	(111)
	Infant mice	Carnitine palmitoyltransferase 1 (CPT1)	Inhibition of CPT1 Reduces Fatty Acid Oxidation and Prevents Cell cycle of cardiomyocytes Arrest	(113)
	Adult mice	Carnitine palmitoyltransferase 1b (CPT1b)	Inhibition of CPT1b Activity Reduces Fatty Acid Oxidation and Re-Enters Cardiomyocytes into the Cell Cycle	(112)
	P14 mice	Carnitine palmitoyltransferase 2 (CPT2)	Inhibition of CPT2 activity increases mouse cardiomyocyte proliferation	(114)
	Adult mice post-MI	Acyl-CoA synthetase long-chain family member 1 (ACSL1)	Knockdown of ACSL1 Enhances Proliferation of Primary Mouse Cardiomyocytes and Myocardial Regeneration in Adult Mice with MI	(115)
	Infant mice	Peroxisome proliferator-activated receptor α (PPAR α)	PPAR α-mediated fatty acid β-oxidation delays Cell cycle of cardiomyocytes arrest in neonatal mice	(113)
	Infant mice, adult mice post-MI and zebrafish	Peroxisome proliferator-activated receptor δ (PPAR δ)	Activation of PPAR*δ* Causes Arrest of the Cardiac Fibroblast Cycle and Reduced Cardiomyocyte Proliferation in Mice After MI; Conversely, Inhibition of PPARδ Reduces Cardiomyocyte Proliferation After Zebrafish Cardiac Injury	(172–174)
	Postnatal mice and adult mice	Sphingosine kinase (SphK)	SphK2 promotes myocardial cell proliferation and regeneration through nuclear S1P dependent histone acetylation regulation, while SphK1 inhibits cardiac fibroblast regeneration through S1P autocrine mechanism	(116)
Glycolysis	Infant mice	Glucose transporter 1 (GLUT1)	GLUT1 Overexpression Increases Glucose Metabolism and Extends the Proliferation Window of Neonatal Mouse Cardiomyocytes	(119)
	Adult mice post-I/R	Phosphofructokinase 2 (PFK2)	PFK2 overexpression enhances contractility in hypoxic mouse hearts	(120)
	Adult mice post-MI	Pyruvate dehydrogenase kinase (PDK4)	Cardiac-specific deletion of PDK4 enhances glycolysis levels and promotes cell cycle re-entry in cardiomyocytes after MI	(111)
	Zebrafish and adult mice post-MI	M2-pyruvate kinase (PKM2)	Overexpression of PKM2 promoted cardiomyocyte proliferation and myocardial regeneration after MI in adult mice. In contrast, deletion of PKM2 prompted zebrafish Cell cycle of cardiomyocytes arrest	(125, 126)
Krebs cycle	Infant mice and adult mice post-MI	Succinate dehydrogenase (SDH)	Malonic acid promotes cell cycle re-entry after MI in adult mice by inhibiting SDH	(128)
	Infant mice and adult mice post-I/R	mitochondrial peroxidase (mCAT)	mCAT overexpression reduces ROS deposition and inhibits cell cycle arrest	(6)
	Infant mice and adult mice post-I/R	N-acetylcysteine (NAC)	Reduction of ROS accumulation by overexpression of NAC inhibits cell cycle arrest	(6)
	Neonatal mice	Paired-like homeodomain 2 (Pitx2)	Pitx2 Knockdown Generates ROS to Promote Cell cycle of cardiomyocytes Arrest in Neonatal Mice	(129)
Amino acid metabolism	Zebrafish and infant mice	Mammalian target of rapamycin (mTOR)	Activation of mTOR Signaling by Elevated Glutamine Prolongs the Cell cycle of cardiomyocytes in Neonatal Mice; Conversely, Inhibition of mTOR Signaling Impairs Cardiac Regeneration in Zebrafish	(136, 138)

### 5.2 Glycolysis reverses cardiomyocyte cell cycle arrest

Glucose serves as a crucial energy source in the heart, where ATP production occurs through cytoplasmic glycolysis and mitochondrial oxidation of pyruvate derived from glycolysis. Glucose enters cardiomyocytes via glucose transporter protein 1 (GLUT1), where it is phosphorylated by hexokinase (HK) and subsequently participates in various metabolic processes. GLUT1 functions primarily as a glucose transporter protein in mammalian embryonic and neonatal hearts (117). Overexpression of GLUT1 in neonatal mouse hearts has been shown to enhance glycolytic efficiency (118) and facilitate cardiac regeneration following cryoinjury (119). These findings provide novel evidence supporting the notion that elevated expression of GLUT1 promotes cardiomyocyte proliferation and cardiac regeneration through enhanced glycolysis.

Key components of glycolysis are involved in the regulation of myocardial regeneration. Phosphofructokinase2 (PFK2), an essential enzyme in glycolysis, has been shown to enhance cardiomyocyte contractility when overexpressed in hypoxic mice (120), suggesting its potential cardioprotective function following cardiac injury. Pyruvate dehydrogenase kinase (PDK), another significant enzyme involved in glycolysis, exhibits significantly increased expression during cardiac development and further upregulation in adult hearts (121), consistent with the pattern of postnatal cardiomyocyte cell cycle arrest. Moreover, both zebrafish cryoinjury and mammalian MI models demonstrate substantial upregulation of PDK within the infarct zone (122, 123). Interestingly, overexpression of PDK3 promotes cardiomyocyte proliferation following zebrafish cardiac cryoinjury, but fails to influence scar repair (122). In contrast, cardiac-specific deletion of PDK4 facilitates cardiomyocyte proliferation and cardiac repair in the injured region after MI by enhancing glycolysis (124). Notably, as isoenzymes of PDK3 and PDK4 exhibit opposing roles in cardiac repair. This discrepancy may be attributed to their distinct distribution patterns, necessitating further investigation into the underlying mechanisms. The rate-limiting enzyme in the final step of glycolysis, pyruvate kinase muscle isoform 2 (PKM2), shows high expression levels in embryonic and neonatal mouse hearts. Overexpression of PKM2 promotes adult cardiomyocyte proliferation and myocardial regeneration by increasing glucose-6-phosphate dehydrogenase expression (125). Deletion of PKM2 inhibits glycolysis and reduces cardiomyocyte proliferation in zebrafish (126). Therefore, targeting specific glycolytic enzymes represents a promising therapeutic strategy for promoting cardiac repair and regeneration.

Collectively, these studies indicate that glycolysis plays a crucial role in regulating cardiomyocyte proliferation and cardiac regeneration following injury. Consequently, targeting glucose metabolism to reverse cardiomyocyte cell cycle arrest emerges as an efficacious strategy for promoting adult cardiac regeneration.

### 5.3 Tricarboxylic acid cycle metabolites regulate cardiomyocyte cell cycle arrest

The postnatal transition from glycolysis to fatty acid oxidation as the primary energy source in mammals is accompanied by a significant increase in mitochondrial abundance, which results in elevated levels of ROS during this period. The increased ROS levels subsequently induce DNA damage, leading to cell cycle arrest in cardiomyocytes (6). Therefore, understanding the role of mitochondrial metabolites in regulating this metabolic switch is crucial for developing research strategies focused on metabolic targeting for adult cardiac regeneration.

On one hand, inhibition of mitochondrial metabolism in cardiomyocytes leads to the reversal of cardiomyocyte cell cycle arrest. Succinate dehydrogenase (SDH) serves as a crucial link between the TAC and oxidative phosphorylation, playing a crucial role in cell cycle regulation and metabolic reprogramming (127). A study demonstrated that malonate-induced inhibition of SDH extended the window for cardiomyocyte proliferation in juvenile mice and promoted cardiomyocyte proliferation, hypertrophy, and cardiac regeneration in adult mice with MI. Notably, this inhibition was accompanied by an enhancement of glucose metabolism and a reduction in TAC cycle metabolism (128), consistent with the metabolic reprogramming process observed during the shift from oxidative phosphorylation to glycolysis in the adult heart, which facilitates cardiomyocyte proliferation and cardiac regeneration.

On the other hand, inhibition of oxidative pathways during mitochondrial metabolism leads to a reduction in ROS production. Martin et al. discovered that activation of antioxidant responses decreased ROS production and facilitated repair following cardiac injury (129). Sadek et al. reported that overexpression of mitochondrial peroxidase (mCAT) and the ROS scavenger, N-acetylcysteine (NAC) system, reduced DDR and promoted cardiomyocyte proliferation (6). Ge et al. demonstrated that Pitx2 deficiency failed to restore neonatal mouse hearts with AR due to the activation of genes associated with ROS scavenging by Pitx2 expression (129). Pei et al. demonstreated that the antioxidant hydrogen sulfide (H_2_S) eliminated ROS and reversed cardiomyocyte cell cycle arrest. Conversely, propylglycine (PAG), an inhibitor of H_2_S synthesis, was found to accumulate ROS, thereby suspending cardiomyocyte proliferation and cardiac regeneration in neonatal mice (130). In contrast, administration of NAD^+^ (NaHS), a hydrogen donor, attenuated H_2_S-mediated ROS accumulation and improved cardiac regeneration through enhanced cardiomyocyte proliferation and elimination of ROS after MI in P7 mice (130). These findings unveil a protective mechanism aimed at reducing mitochondrial oxidative stress-induced cardiomyocyte cell cycle arrest while providing a novel therapeutic strategy for HF.

The mitochondrial pyruvate carrier (MPC), located on the inner mitochondrial membrane, is responsible for transporting pyruvate from the cytoplasm to the mitochondrial matrix, where it participates in the tricarboxylic acid cycle, gluconeogenesis, and metabolism of lipids and amino acids to provide energy for the organism. The MPC plays a key role in the proliferation of cardiomyocytes and in the regulation of the cell cycle. Studies have shown that MPC1 deficiency leads to cardiomyocyte hypertrophy and heart failure, whereas MPC1 overexpression attenuates the hypertrophic response of cardiomyocytes (131). In addition, drugs modulating MPC activity may promote cardiomyocyte survival and functional recovery by improving energy metabolism and antioxidant capacity of cardiomyocytes. Currently, the MPC inhibitor UK5099 has been used to investigate its therapeutic potential in metabolic diseases (132), so could drugs modulating MPC activity further influence the cell cycle by regulating the metabolic level of cardiomyocytes, which could ultimately be applied in the clinical treatment of cardiovascular diseases such as MI?

Taken together, these studies provide compelling evidence for the crucial role of mitochondrial metabolites in modulating cardiac metabolism and highlight their promising therapeutic potential in facilitating adult cardiac regeneration (Table 1).

### 5.4 Amino acid oxidation promotes cardiomyocyte cell cycle arrest

Proteins play a pivotal role in the growth and maturation of cardiomyocytes, with enhanced protein synthesis leading to increased amino acid metabolism. To gain further insights into the involvement of amino acid oxidation in cardiac regeneration, studies have primarily focused on branched chain amino acids (BCAAs), namely leucine, isoleucine, and valine. BCAA levels exhibited dynamic fluctuations during mouse heart development. Specifically, valine, leucine, and isoleucine showed an increase during the postnatal phase, reaching a peak at day 9, followed by a decline in adulthood. Furthermore, most of the differentially expressed genes is directly associated with BCAA concentrations (133), indicating that amino acid metabolism plays an essential role in cardiomyocyte maturation. Therefore, we hypothesized that inhibiting enzymes involved in the catabolic pathway of BCAAs can enhance cardiomyocyte proliferation.

The mammalian target of rapamycin (mTOR) signaling pathway plays a critical role in cardiac development and growth (134, 135). Increased expression of glutamine, an amino acid transporter, in zebrafish and neonatal mouse cardiomyocytes activates the mTOR signaling pathway and regulates cardiomyocyte proliferation. Moreover, high concentrations of leucine and glutamine have been found to activate mTOR signaling and facilitate cardiac regeneration (136). Therefore, it is worth investigating whether the concentration of BCAAs also impacts the proliferative capacity of cardiomyocytes. Previous study has demonstrated that BCAAs activate the mTOR signaling pathway and induce metabolic reprogramming from fatty acid oxidative metabolism to glycolysis via HIF-1α (137), which is consistent with the metabolic reprogramming observed during cardiac regeneration. Conversely, inhibition of mTOR signaling blocks cardiac regeneration in zebrafish and promotes maturation of human pluripotent stem cell (iPSC)-derived cardiomyocytes (138, 139). Thus, it is evident that cardiomyocyte amino acid metabolism influences both cardiomyocyte proliferation and cardiac regeneration. However, further comprehensive investigations are warranted due to limited findings in this research area.

In summary, the blockage of metabolic regulatory mechanisms of the cardiomyocyte cycle represents an important research direction in cardiac regenerative medicine. Future studies will explore the regulatory mechanisms of cardiomyocyte regeneration in depth through metabolic intervention, epigenetic regulation, and multi-omics technology, thereby providing new strategies and methods for the treatment of cardiac diseases.

## 6 Drug development for cell cycle regulation and clinical treatment of CVDs

In recent years, despite significant advancements in the field of CVD treatment, there is an urgent need for novel therapeutic interventions. Among these potential options, targeting cell cycle regulation through drug development holds promise as a new strategy for CVD treatment. The following section provides a comprehensive summary of recent studies in drug discovery and clinical management of CVDs (Table 2), aiming to open new horizons in this field.

**Table 2:** Development of drugs targeting cardiomyocyte cell cycle.

Type	Drugs	Mechanism of action	References
Metabolic drug	Trimetazidine	Trimetazidine exerts a positive effect in regulating the Cell cycle of cardiomyocytes process by inhibiting excessive glycolysis, increasing aerobic oxidation of glucose, and inhibiting lactate accumulation	(142, 159, 160)
	Ranolazine	Ranolazine prevents fatty acid oxidation, favors sugar utilization, and alters the course of the Cell cycle of cardiomyocytes in the failing heart	(175)
	Leucovorin	Leucovorin promotes the transport of long-chain fatty acids into the mitochondrial matrix and regulates the Cell cycle of cardiomyocytes by reducing oxidative stress	(176)
	Glucose-insulin-potassium (GIK)	GIK promotes glycolysis to regulate Cell cycle of cardiomyocytes progression by decreasing free fatty acid levels in the circulation of MI patients	(177)
	CoQ10	CoQ10 improves cardiomyocyte proliferation efficiency during myocardial ischemia by exerting antioxidant effects and reducing ROS release and DDR	(177–179)
	Nicotinamide adenin (NAD+)	NAD+ Regulates Metabolic Levels by Participating in Redox Reactions to Reverse the Cell cycle of cardiomyocytes	(147–151)
ncRNA drug	CDR132l	CDR132l can improve the degree of HF, restore cardiomyocyte function and reverse Cell cycle of cardiomyocytes arrest within a reasonable dose range	(155)

### 6.1 Metabolism-related drugs in CVD treatment

Myocardial energy metabolism plays a crucial role in regulating various pathological processes of CVDs, and interventions targeting myocardial energy metabolism hold promise as the next frontier for CVD prevention and treatment (140, 141).

Trimetazidine is used as an adjunct in the clinical treatment of HF to protect cardiomyocyte energy metabolism under hypoxic or ischemic conditions by reducing fatty acid beta oxidation and promoting glucose metabolism. In rat heart following I/R injury, trimetazidine has been shown to inhibit excessive glycolysis and enhance aerobic glucose oxidation (142). Consequently, trimetazidine exerts favorably influences the regulation of cardiomyocyte cell cycle through modulation of energy metabolism in the injured myocardium. Mitochondria, as the primary site of energy metabolism, have made the development of drugs targeting mitochondrial function regulation a major focus in the treatment of CVDs (143, 144). CoQ10, a naturally occurring compound in the human body closely associated with cardiac energy metabolism, exhibits notable antioxidant properties and effectively reduces the risk of cardiac morbidity (145). Nicotinamide adenine dinucleotide (NAD^+^), also known as coenzyme I, is a crucial central metabolite involved in redox reactions (146). Evidence from animal experiments has demonstrated that NAD^+^ levels are downregulated following cardiac I/R injury, and NAD+ supplementation reduces infarct size while reversing cardiomyocyte cell cycle arrest by regulating metabolic reprogramming (147–151). In recent years, NAD^+^ mediated myocardial metabolic disorders have been recognized as one of the pathogenic mechanisms underlying CVDs. Therefore, exploring the pharmacological role of NAD^+^ holds great promise as a therapeutic avenue.

Enhancing the energy supply through metabolism-related pathways plays a beneficial and complementary role in CVD treatment. Meanwhile, ensuring the judicious and rational utilization of myocardial metabolism-targeted drugs to maintain the homeostasis of energy metabolism represents a crucial next step in CVD drug discovery and development.

### 6.2 NcRNA drugs for the treatment of CVDs

As ongoing research progresses, an increasing number of evidence indicates that ncRNAs play a crucial role as regulatory molecules in modulating the cardiomyocyte cell cycle (152). In recent years, the development of multiple RNA-based therapies has further accelerated advancements in ncRNA-targeted drug discovery and development.

MiRNAs have emerged as promising targets for novel CVD-targeted therapeutics due to their short length, strong conservation, and high stability (153, 154). Currently, CDR132l, an antisense oligonucleotide drug targeting miR-132, has become the first therapeutic drug for HF and entered clinical trials. In a porcine model of HF after MI, CDR132l demonstrated significant improvements in HF severity, restoration of cardiomyocyte function, and reversal of cardiomyocyte cell cycle arrest within a reasonable dosage range (155). MRG-110, an antisense oligonucleotide drug for the treatment of cardiovascular diseases, promotes the growth of new blood vessels by inhibiting miR-92a, thereby exerting a therapeutic effect in accelerating wound healing (156). However, its clinical studies related to CVDs remain limited. Collectively, miRNA-based drugs hold promising prospects for treating MI and promoting myocardial cell cycle recovery following ischemic injury, thus representing novel biological targets worthy of further exploration in CVD treatment.

In comparison to miRNAs, lncRNAs and circRNAs have received relatively less attention in the field of CVD drug discovery and development owing to their limited *in vivo* stability, larger molecular size, and propensity to induce an immune response. Therefore, overcoming these challenges represents the subsequent crucial step for drug developers.

In summary, cell cycle regulation and metabolic regulation are important in the treatment of cardiovascular diseases. Future research will develop more effective and safer therapeutic drugs and methods through targeted therapy, nucleic acid-based therapeutic strategies, metabolic interventions, and clinical translation, providing new hope for the treatment of cardiovascular diseases.

## 7 Conclusion

Nowadays, CVD has become the leading cause of mortality worldwide, posing a severe threat to human life and health. Therefore, there is an urgent need for effective therapeutic strategies for CVD. Currently, the primary means of treating CVD in clinical practice is interventional therapy, which fails to achieve the renewal and regeneration of cardiomyocytes. The limited proliferative capacity of mature cardiomyocytes can be attributed to their terminal differentiation status and subsequent cell cycle arrest. Therefore, promoting cardiac regeneration by modulating cell cycle factors may represent a key strategy for curing cardiomyopathy in the future. In this review, we summarize the current state of research on myocardial regeneration and the mechanisms underlying cardiomyocyte cell cycle arrest, including recent findings and ongoing controversies.

The entry of cardiomyocytes into the cell cycle for division and proliferation represents the primary step in cardiac regeneration. To clearly delineate the critical role of cell cycle-related factors in cardiac regeneration, we mapped the regulators of cell cycle arrest before and after the proliferation window of cardiomyocytes (Figure 3). These regulators include both coding and noncoding genes, and as well as signaling pathways that regulate cardiomyocyte cell cycle exit or re-entry mainly by targeting cyclin-dependent factors. Notably, these regulators function independently, but instead form a complex network that orchestrates cell cycle homeostasis. Given this complexity, further elucidating the mechanisms of these pathways during homeostasis, disease, and regeneration *in vivo* is a crucial step toward targeting these pathways for therapeutic development.

The regulation of the cell cycle by metabolic reprogramming has been extensively studied in the context of tumor cells, while studies focusing on cardiomyocytes, which exhibit limited proliferative capacity, remain underexplored but hold immense potential. In this review, we summarize the current state of research on how myocardial metabolism regulates cell cycle arrest (Table 1). On this basis, modulating relevant metabolic pathways to promote cardiomyocyte cell cycle re-entry, proliferation, and regeneration may eventually evolve into an effective treatment for CVDs. However, several key questions require further investigation. For example, metabolic changes influence cardiomyocyte cell cycle progression; what are the underlying mechanisms by which these metabolic alterations ultimately affect cardiomyocyte proliferation and cardiac regeneration? Additionally, both cell cycle factors and metabolic pathways are involved in cardiac regeneration; are their interactions critical to cardiac regeneration?

In the clinical management of CVDs, drug therapy for cardiomyopathy has guided the direction of clinical research. Drugs such as trimetazidine, ranolazine, and CDR132l have been investigated in clinical trials for their ameliorative effects on cardiomyopathy (Table 2). In basic research, some of these drugs target cardiomyocyte cell cycle processes to exert their functions. For instance, malic acid in cardiac basal fluid exhibits antioxidant activity, scavenges ROS, and regulates cardiomyocyte cell cycle re-entry (157); trimetazidine affects cardiomyocyte cell cycle progression by regulating energy metabolism levels (142, 158–160). Therefore, it is essential to harness the pharmacological potential of validated pathways as drug candidates for inducing cardiac regeneration in adults. However, further exploration is required to determine the the population, side effects, and optimal dosage of therapeutic agents for cardiomyopathy (161).

So far, several key questions remain unresolved. It is well established that vascular endothelial growth factor (VEGF) binds to its receptor to promote vascular regeneration (162). However, the nascent vascular endothelium formed after MI has been shown to contribute to cardiomyocyte proliferation (163). It remains unclear whether VEGF, a well-characterized therapeutic factor for MI (164), can directly modulate cardiomyocyte cell cycle progression. Cardiac fibroblasts, which constitute the major cellular component of the heart, have previously been demonstrated to reprogrammed into cardiomyocytes through the action of specific transcription factors (165). In addition, cardiomyocytes can interact with endothelial cells and immune cells to influence the cell cycle and thus their proliferative potential (93, 166). However, lymphocytes also play an important role in cardiac inflammation and immune responses. So, do cardiomyocytes interact with lymphocytes to regulate cardiomyocyte proliferation? Stem cell therapy would be a potential cardioprotective strategy after MI (167). Previous studies have demonstrated that stem cells can act as immunomodulators and promote myocardial regeneration through anti-inflammatory effects (168, 169). However, the ability of stem cell therapies to reprogram cardiomyocyte cell cycle progression through the interaction of other cellular components (fibroblasts, endothelial cells, and platelet-derived growth factors, among others) remains to be further explored.

Currently, some progress has been made in the clinical treatment of myocardial regeneration. Canseco et al. found that cardiac load reduction by left ventricular assist device (LVAD) can induce the proliferation of cardiomyocytes (170). In addition, positive progress has been made in cell therapy trials, providing an important reference for the development of cardiac regenerative medicine (170). However, research on cardiomyocyte proliferation and regeneration still faces many challenges. For example, most of the current studies are in the stage of animal experiments, and the safety and efficacy of some of the technologies in human beings have yet to be further verified. In addition, the mechanism of cardiomyocyte proliferation and regeneration is complex and involves multiple cell types and signaling pathways, so how to accurately regulate the proliferation and regeneration of cardiomyocytes is still an urgent problem to be solved.

Despite some basic science advances in cardiac regeneration research, a number of controversies and questions remain. There is wide controversy in the academic community regarding the existence and function of cardiac stem cells. Early studies suggested that cardiac stem cells (e.g., c-Kit+ cells, Sca-1+ cells, etc.) have significant regenerative potential, but recent findings suggest that these cells have a very limited ability to differentiate into cardiomyocytes *in vivo* (171). Therefore, further studies are needed to investigate whether cardiac stem cells exist and the specific mechanisms of their role in cardiac regeneration. In addition, current studies have not fully elucidated whether cardiomyocyte regeneration is mainly dependent on the dedifferentiation of cardiomyocytes or on the reactivation of progenitor/stem cells in the heart, which is still controversial (170). Therefore, researchers need to further investigate the specific mechanisms of cardiomyocyte regeneration and how to promote endogenous cardiomyocyte regeneration by regulating cell cycle and metabolic pathways. Overall, the field of cardiac regeneration has made remarkable scientific progress, but there are still many problems, limitations, and research gaps. Future research needs to bridge the gap between basic science and clinical applications to provide a more solid theoretical foundation and feasible clinical protocols for cardiac regenerative therapies.

In this review, we systematically sort out the critical role of the cell cycle in cardiomyocyte proliferation and cardiac regeneration, discuss the precise regulation of the cell cycle by cell cycle regulators and metabolism, and further review the development and clinical applications of cell cycle-related drugs for cardiovascular diseases. This information will contribute to the treatment of cardiovascular diseases and provide promising directions for future research in the treatment of cardiovascular diseases, which will facilitate the exploration and utilization of new therapeutic strategies. In conclusion, we are optimistic that impressive progress in the direction of human cardiac regeneration is possible.
